# Neutrophil Extracellular Traps in ANCA-Associated Vasculitis

**DOI:** 10.3389/fimmu.2016.00256

**Published:** 2016-06-30

**Authors:** Daniel Söderberg, Mårten Segelmark

**Affiliations:** ^1^Department of Medical and Health Sciences, Linköping University, Linköping, Sweden; ^2^Department of Nephrology, Linköping University, Linköping, Sweden

**Keywords:** neutrophil extracellular traps, ANCA-associated vasculitis, ANCA, NET, small-vessel vasculitis, NET remnants

## Abstract

A group of pauci-immune vasculitides, characterized by neutrophil-rich necrotizing inflammation of small vessels and the presence of antineutrophil cytoplasmic antibodies (ANCAs), is referred to as ANCA-associated vasculitis (AAV). ANCAs against proteinase 3 (PR3) (PR3-ANCA) or myeloperoxidase (MPO) (MPO-ANCA) are found in over 90% of patients with active disease, and these ANCAs are implicated in the pathogenesis of AAV. Dying neutrophils surrounding the walls of small vessels are a histological hallmark of AAV. Traditionally, it has been assumed that these neutrophils die by necrosis, but neutrophil extracellular traps (NETs) have recently been visualized at the sites of vasculitic lesions. AAV patients also possess elevated levels of NETs in the circulation. ANCAs are capable of inducing NETosis in neutrophils, and their potential to do so has been shown to be affinity dependent and to correlate with disease activity. Neutrophils from AAV patients are also more prone to release NETs spontaneously than neutrophils from healthy blood donors. NETs contain proinflammatory proteins and are thought to contribute to vessel inflammation directly by damaging endothelial cells and by activating the complement system and indirectly by acting as a link between the innate and adaptive immune system through the generation of PR3- and MPO-ANCA. Injection of NET-loaded myeloid dendritic cells into mice results in circulating PR3- and MPO-ANCA and the development of AAV-like disease. NETs have also been shown to be essential in a rodent model of drug-induced vasculitis. NETs induced by propylthiouracil could not be degraded by DNaseI, implying that disordered NETs might be important for the generation of ANCAs. NET degradation was also highlighted in another study showing that AAV patients have reduced DNaseI activity resulting in less NET degradation. With this in mind, it might be that prolonged exposure to proteins in the NETs due to the overproduction of NETs and/or reduced clearance of NETs is important in AAV. However, not all ANCAs are pathogenic and some might possibly also aid in the clearance of NETs. A dual role for ANCAs in relation to circulating NET levels has been proposed because a negative correlation was observed between PR3-ANCA and NET remnants in patients in remission.

## Antineutrophil Cytoplasmic Antibody-Associated Vasculitis

Vasculitides are inflammations in the walls of blood vessels, and they can affect any organ system in the body. They are divided into broad groups based on the size of the vessels predominantly being affected. A subgroup of small-vessel vasculitides is characterized by a scarcity of immune depositions (pauci-immune) and the presence of antineutrophil cytoplasmic antibodies (ANCAs) and is referred to as ANCA-associated vasculitis (AAV) (1). AAV comprise three diseases, including granulomatosis with polyangiitis [GPA, previously known as Wegener’s granulomatosis (2)], microscopic polyangiitis (MPA), and eosinophilic granulomatosis with polyangiitis (EGPA, previously known as Churg–Strauss syndrome) (3). GPA and EGPA share the feature of necrotizing granulomatous inflammation of the lower respiratory tract, whereas MPA is characterized by the absence of this component. Also, GPA often affects the upper respiratory tract and can result in rhinitis, otitis, and cartilage destruction, while eosinophilia and asthma are defining features of EGPA. Renal involvement is observed in as many as 90% of the patients with MPA, compared to 80% of the patients with GPA and 45% in EGPA. All three diseases affect the skin, joints, eyes, and nerves to various extents (1, 4). There is also an increased incidence of venous thromboembolism in AAV patients, especially during active disease (5, 6). AAVs are relapsing–remitting diseases, and 50% of the patients have a relapse within 5 years of successful treatment. The mortality rate is around 80% at 1 year when left untreated, but with current treatments, the mortality rate is reduced to 25% within 5 years (7).

Autoantibodies specific for proteinase 3 (PR3) (PR3-ANCA) or myeloperoxidase (MPO) (MPO-ANCA) are found in over 90% of patients with active disease (8), and these are important as diagnostic tools. The association between PR3- and MPO-ANCAs and active disease in AAV suggests a pathogenic role for the autoantibodies, and such a role is supported by results from animal models (9, 10) and *in vitro* studies showing that PR3- and MPO-ANCAs can activate neutrophils to produce reactive oxygen species (ROS) and proteolytic enzymes (11). ANCA-induced neutrophil activation also leads to increased adhesion of the neutrophils (12) and the activation of the alternative complement pathway (13) with the generation of C5a. C5a in turn potentiates the inflammatory response by priming neutrophils and acting as a chemoattractant to recruit more neutrophils to the inflammatory site (14). However, ANCA levels do not conclusively predict relapses (15, 16), and there is an unmet need for biomarkers for this purpose.

## Neutrophil Extracellular Traps

**Figure 1:**
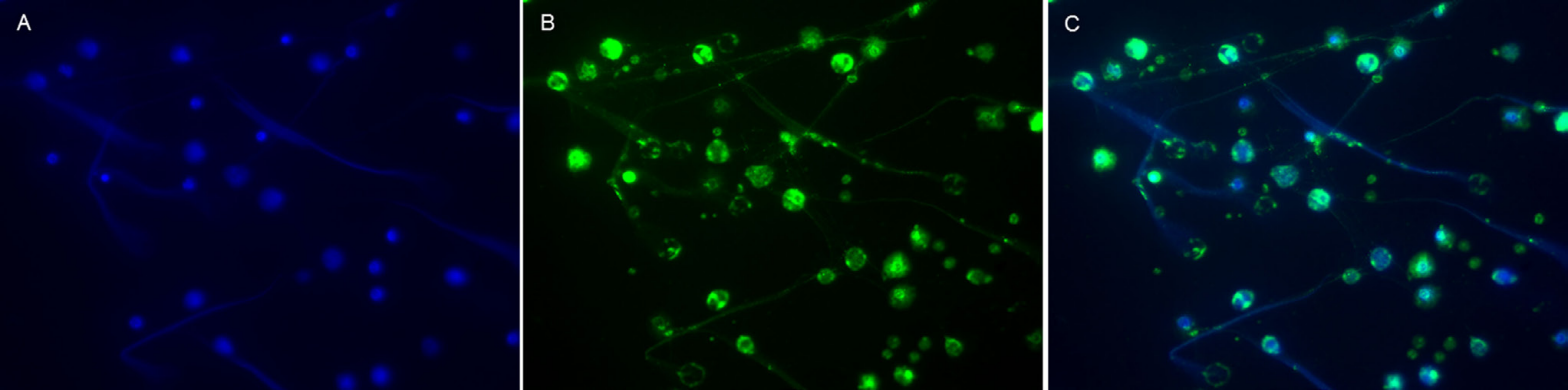
**Visualization of MPO in NETs from human neutrophils**. Neutrophils isolated from human peripheral whole blood were cultured for 4 h at 37°C with 25 nM PMA. NETs were then visualized by immunofluorescence microscopy using a 40× objective. **(A)** DNA, the backbone of NETs, was labeled with DAPI (blue). **(B)** MPO (clone 2B11), a granulae protein within the NETs (17), was labeled with a Dylight 488-conjugated antibody (green). **(C)** DNA and MPO (merged) co-localized in the NETs. NETs, neutrophil extracellular traps; PMA, phorbol-12-myristate-13-acetate. Blood samples were collected after obtaining informed consent in accordance with the declaration of Helsinki, and the study was approved by the Regional Ethical Review Board in Linköping. This figure is not intended to be quantitative, but only to serve as a representative image of common prior knowledge regarding the presence of MPO in NETs (17).

Neutrophil extracellular traps (NETs) were first described in 2004 as a means for neutrophils to trap and kill bacteria (17) and are released as a result of a programed cell death mechanism referred to as NETosis (18, 19). NETs consist of a DNA backbone and various proteins with proinflammatory characteristics, such as histones, high-mobility group box 1 (HMGB1), LL37, neutrophil elastase (NE), calprotectin (S100A8/S100A9, MRP8/14), and, interestingly, MPO (Figure 1) and PR3 (20, 21). All described ANCA antigens are components of NETs. NETosis depends on a cascade of events that lead to the mixing of nuclear, cytoplasmic, and granular components before the NETs are released into the surrounding matrix (18). NETosis has been shown to depend on NADPH oxidase and ROS production as well as on autophagy and histone citrullination. Peptidyl arginine deiminase 4 (PAD4), NE, and MPO have been shown to play important roles in this signaling pathway (18, 22, 23). More recently, other forms of NETosis have also been described, including NETosis with the release of mitochondrial DNA (mtDNA ETs) (24) instead of nuclear DNA and ROS-independent NETosis (25–27). Interestingly, when releasing mtDNA ETs, the neutrophils can also remain viable (24). In addition to their role as antimicrobial agents, NETs of both nuclear and mitochondrial origin have also been connected to various autoinflammatory and autoimmune diseases (28–33).

## NETs are Present in Glomeruli, Skin Lesions, and Thrombi of AAV Patients

Dying neutrophils surrounding the walls of small vessels are a histological hallmark of AAV. Traditionally, it has been assumed that these neutrophils die by necrosis, but in 2009, Kessenbrock et al. showed that NETs were present in the glomeruli in kidney biopsies from AAV patients (34). They reported the presence of NETs as co-localizations of DNA, histones, and the granule proteins PR3, LL37, NE, and MPO in various combinations (34). This phenomenon was later confirmed by others (35–38). Although the method for detecting NETs in glomeruli was rather similar in these studies, i.e., visualization of DNA and histones (although some looked at citrullinated histones) in combination with the granule proteins already described – one study also reported the presence of PAD4 in the NETs, which is necessary for histone citrullination (35), and another study detected LAMP2, which is also an ANCA antigen (36, 39).

Neutrophil extracellular traps have also been shown to be present in skin lesions (40, 41) and thrombi from AAV patients (38, 42). In the studies investigating NETs in skin lesions, the presence of NETs was based on extracellular MPO (40, 41) or on DNA in combination with MPO (41). The presence of NETs in thrombi was defined not only as co-localizations of DNA and MPO but also as citrullinated histones alone (38). Another study also defined NETs based only on the presence of citrullinated histones (42).

## Increased Levels of NETs and NET-Associated Proteins in the Circulation of AAV Patients

In addition to the presence of NETs in various lesions from AAV patients, it has been shown that these patients also have elevated levels of NETs in the circulation (34, 43–46) (Table 1). In these studies, NETs were defined as nucleosome/MPO complexes (34, 43, 46), total DNA or DNA/MPO or citrullinated histone 3 (H3) complexes (45), DNA/histone complexes (46), or as nuclear DNA or mtDNA (44). There are also several observations regarding circulating neutrophil components that are the main constituents of NETs. Important examples are HMGB1, calprotectin (S100A8/S100A9, MRP8/14), PR3, MPO, and NE (46–55) (Table 1). The study measuring calprotectin used longitudinally collected samples from the NORAM trial and found that calprotectin levels correlated with disease activity (47), and the studies measuring NE observed a correlation between NE and Birmingham Vasculitis Activity Score (i.e., disease activity) (51). However, the presence of these proteins in the circulation does not reveal whether they are released as a result of NETosis or by other mechanisms, but it was recently shown that the levels of MPO and NE correlate with the levels of DNA/MPO complexes in the circulation (46). The capability of using NETs as a biomarker to monitor disease activity in AAV has not been evident in previous studies. No study has so far measured the levels of NETs longitudinally in patients at multiple time points. In some cross-sectional studies, the levels of NETs have been measured in patients during both remission and active disease, but with inconclusive results regarding their correlation with disease activity (43, 45).

**Table 1:** **NET-associated proteins and structures present in the circulation of AAV patients**.

Protein/structure	Method	AAV vs. HC	Correlation with disease activity
Nucleosome + MPO complexes	ELISA	+ (34, 43, 46)	Yes (34, 43, 46)
DNA + MPO or citrullinated histone 3 complexes	ELISA	+ (45)	No (45)
DNA + histone complexes	ELISA	+ (46)	No (46)
DNA	PicoGreen	+ (45)	No (45)
mtDNA	qPCR	+ (44)	Yes (44)
Nuclear DNA	qPCR	+ (44)	No (44)
PR3	ELISA/Luminex	+ (46, 49, 52, 53, 55)	No (46)
MPO	ELISA	+ (53)	Yes (46)
HMGB1	Western blot/ELISA	+ (48, 50, 54)	Yes (48, 50, 54)
Calprotectin	ELISA	+ (47)	Yes (47)
NE	ELISA/Luminex	+ (46, 51)	Yes (46, 51)

*Numbers in parenthesis indicate referenced publication*.

*AAV ANCA-associated vasculitis; HC, healthy blood donors; +, increased levels; nd, not determined; PR3, proteinase 3; HMGB1, high-mobility group box 1 protein; MPO, myeloperoxidase; mtDNA, mitochondrial DNA; nDNA, nuclear DNA; NE, neutrophil elastase*.

## Proinflammatory Aspects of NETs in AAV

Neutrophil extracellular traps have previously been described as double-edged swords of innate immunity (56), considering that they are involved in both fighting pathogens and in contributing to autoinflammatory and autoimmune conditions. Various proinflammatory aspects of NETs in general might also be important in the pathogenesis of AAV. For example, NETs can cause endothelial damage (57–59) and can activate the alternative complement pathway (60), which, as already mentioned, plays an important role in amplifying the inflammatory process in AAV. Further, anti-histone antibodies have been shown to ameliorate experimental glomerulonephritis, emphasizing the proinflammatory aspect of histones in the NETs (61). It has also been shown that the presence of histones in NETs can contribute to thrombus formation (62) and that the presence of tissue factor (63, 64) in NETs can contribute to the generation of thrombin. In turn, it has been demonstrated that activated platelets can stimulate neutrophils to release NETs and that platelet-induced NETs propagate deep vein thrombosis in mice (65). Others have shown that HMGB1 expressed on platelets mediate the formation of platelet-induced NETs and that this process is dependent on autophagy (66), and in mice, it has been shown that platelet-derived p-selectin can induce NETosis (67). Increased levels of platelet-neutrophil aggregates and soluble P-selectin have been observed in the circulation of AAV patients during active disease and to correlate with disease activity (46). Additionally, HMGB1 has also been shown to potentiate the effect of ANCAs on NET formation (68). Oxidized mtDNA ETs released from neutrophils in systemic lupus erythematosus (SLE) have been shown to possess proinflammatory characteristics (33), and the role of mtDNA in general as a danger-associated molecular pattern has been extensively described (69).

## Spontaneous NET Formation *In Vitro*

Earlier studies have shown that neutrophils from AAV patients are less prone to undergo apoptosis (70), suggesting that these neutrophils are more prone to other forms of cell death. Indeed, *in vitro* studies have shown that neutrophils from AAV patients are more prone to release NETs spontaneously than neutrophils from healthy blood donors (36, 43, 71). A subpopulation of neutrophils, referred to as low-density granulocytes (LDGs), have been shown to spontaneously release NETs significantly more than normal-density neutrophils, and these LDGs haves been proposed to be the major source of NETs in AAV (71). However, the same study also showed that normal-density neutrophils from AAV patients spontaneously released more NETs than normal-density neutrophils from healthy blood donors (71). More detailed studies of LDGs in SLE have revealed that LDGs express increased levels of mRNA of various immunostimulatory bactericidal proteins and alarmins compared to normal-density neutrophils (59). It is important to note that during the various isolation procedures normally used to obtain neutrophils from peripheral whole blood, LDGs will not be included because they will be found in the fraction of peripheral blood mononuclear cells. This is important to consider in future *in vitro* studies of neutrophils and NET formation.

## ANCAs as Mediators of NETosis

**Figure 2:**
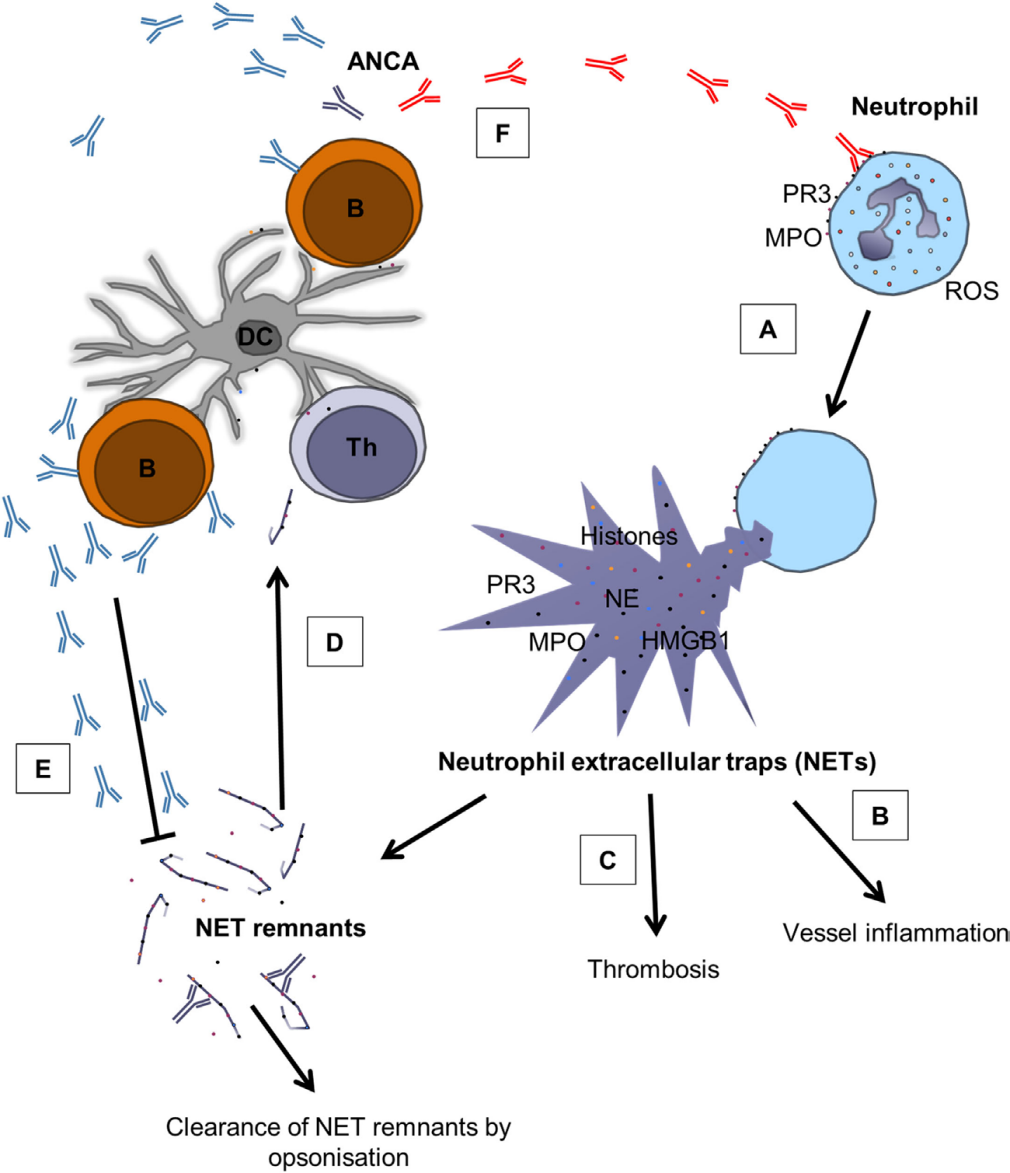
**The role of NETs in AAV and the complex relation between ANCAs and NETs**. **(A)** Pathogenic ANCAs (red) reacting with PR3 and MPO on the surface of neutrophils cause ROS production and the release of NETs through NETosis (34, 78, 79). **(B)** NETs contain various proinflammatory mediators, such as histones, HMGB1, PR3, MPO, and NE (17, 20, 21), and contribute to vessel inflammation by damaging endothelial cells (57–59) and by activating the complement system (60). **(C)** NETs do also promote thrombosis through the expression of histones (62) and tissue factor (63, 64). **(D)** NETs can also act as a link between the innate and adaptive immune system through the generation of ANCAs (41, 80). **(E)** ANCAs seem to belong to repertoire of “natural” antibodies (81), indicating that not all ANCAs are pathogenic, and it has been proposed that ANCAs can aid in clearance of circulating NET remnants (43). **(F)** However, under unfavorable circumstances, pathogenic ANCAs (red) are produced, creating a vicious circle that promotes inflammation. B, B cell; Th, T helper cell; DC, dendritic cell. Modified from Ref. (43) with permission from Oxford University Press.

In addition to the effects already ascribed to PR3- and MPO-ANCA in terms of neutrophil activation, they are also capable of inducing NETosis (Figure 2) (34). Although the exact mechanism for neutrophil activation by ANCAs is not clear, full activation requires binding of autoantibodies to both Fc-receptors and to PR3/MPO on the surface of neutrophils (72). It has been suggested that neutrophil activation, in this case evaluated as ROS production by MPO-ANCA, is epitope-specific, that epitope specificity varies with disease activity and that ANCAs activate neutrophils more robustly during active disease (73). Furthermore, *in vitro* studies have shown that neutrophils from patients are more easily activated (they produce more ROS) by ANCAs (in this case PR3-ANCA) than neutrophils from healthy blood donors (74). It has previously been shown that neutrophils from AAV patients possess increased membrane expression of PR3 (75, 76), which could possibly be explained by disrupted epigenetic silencing of the PR3 and MPO gene in these patients (77). However, in the study by Ohlsson et al. the results could not be explained by increased PR3 expression on the cell surface of neutrophils from patients or the ANCA levels (74). Rather, epitope specificity and affinity seemed to be of importance for the antibodies’ ability to activate neutrophils (74). It has also been shown that MPO-ANCA has higher affinity for MPO during active disease and that MPO-ANCA induces more NETs during active disease (78), and the observation that the affinity for MPO-ANCA is important for the ability to induce NETs was recently confirmed by another group (79). In summary, it seems that both epitope specificity and affinity are important for neutrophil activation by ANCAs and that at least the affinity is important for their ability to induce NETs.

## NETs: Bridging Innate and Adaptive Immunity

It has been shown using NETotic neutrophils from mice that MPO and PR3 can be taken up from the NETs by myeloid dendritic cells (mDCs) and that injection of NET-loaded mDCs into mice results in circulating MPO- and PR3-ANCA and development of AAV-like disease (41). The addition of DNaseI to the *in vitro* cultures prevented PR3 and MPO uptake by the mDCs from the NETs, and when mice were injected with those mDCs, the mice did not develop disease (41). In the same study, injection of mDCs cocultured with apoptotic neutrophils into mice also caused autoantibody production, but those mice did not develop AAV-like disease. These experiments indicate that NETs show higher immunogenicity than apoptotic cells and that the structural integrity of the NETs is important for transferring NET-antigens to mDCs and the subsequent production of pathogenic autoantibodies. This is in line with a previous study showing that rats immunized with apoptotic neutrophils do develop ANCAs, but not disease (82). In another study, rats were immunized with NETs induced by phorbol-12-myristate-13-acetate (PMA) and propylthiouracil (PTU) (which together induced abnormal NETs that could not be degraded by DNaseI) or were given PTU orally in combination with PMA (intraperitoneal injection), and these rats developed MPO-ANCA and pulmonary capillaritis or glomerulonephritis and pulmonary capillaritis, respectively (80). This resembles the situation in humans, where over 20% of patients with Graves’ disease treated with PTU develop MPO-ANCA and some also AAV-like disease (83, 84).

## NET Formation vs. Clearance: The Importance of Balance

The studies described earlier imply that NETs can act as a link between the innate and adaptive immune system with the production of pathogenic ANCAs. With this in mind, it might be that prolonged exposure to the proteins in the NETs due to the overproduction of NETs and/or reduced clearance of NETs is important in AAV. In line with this, it has been shown that AAV patients have reduced capacity to degrade NETs *in vitro* (78). This could possibly be due to the reduced DNaseI activity observed in these patients compared to healthy blood donors, although DNaseI activity did not correlate with disease activity. Thus, the elevated levels of NETs in the circulation of AAV patients might also be explained by the reduced capacity to clear the NETs from the circulation. Interestingly, low levels of both PR3-ANCA and MPO-ANCA can be found in the circulation of healthy individuals (81), indicating that the presence of ANCAs does not necessarily lead to disease development. Rather, ANCAs might be part of the repertoire of natural antibodies that are important for maintaining homeostasis (85). In line with this, a dual role for ANCAs was recently suggested, where the autoantibodies in addition to inducing NET formation can also aid in the clearance of NETs (Figure 2) (43), possibly through opsonization and the formation of immune complexes. This hypothesis was proposed because a negative correlation was observed between PR3-ANCA and circulating NET remnants in AAV patients in remission (43). As others have shown that the pathogenicity of ANCAs seems to vary with both epitope specificity (73) and affinity (78) and that these parameters change with disease activity, it appears that ANCAs might play different roles at different stages of AAV. Together, these studies might suggest how and why all individuals can possess ANCAs but only some develop AAV.

## Infections and ANCAs

Antineutrophil cytoplasmic antibodies are common in chronic infections, such as *Pseudomonas aeruginosa* infections, in patients with cystic fibrosis, tuberculosis, HIV, and infective endocarditis (86–89). Infections are also implicated in the pathogenesis of AAV and as a trigger of relapses. Molecular mimicry, either directly (90) or indirectly through autoantigen complementarity (91), is the traditional way to explain the relationship between AAV and infection. However, infections lead to neutrophil activation, which triggers NETosis. Lipopolysaccharide-activated platelets can also activate neutrophils to release NETs (92), and this suggests an indirect way in which bacteria can contribute to NETosis as well as to the coagulation cascade and thrombosis formation discussed earlier. In sepsis, the liver sinusoids are filled with neutrophils undergoing NETosis (93), and in infective endocarditis, a role for NETs has also been described (94). ANCAs are found in up to 20% of patients with endocarditis (95), and many of these patients have symptoms resembling vasculitis, such as fever, increased CRP, weight loss, malaise, multiform skin lesions, and renal involvement (1, 96–98).

## Concluding Remarks/Discussion

This review has outlined the role of NETs in the pathogenesis of AAV. There is compelling evidence that NETs contribute to vessel inflammation directly by damaging endothelial cells and by activating the complement system and indirectly by acting as a link between the innate and adaptive immune system through the generation of PR3-ANCA and MPO-ANCA. This can lead to a vicious circle because ANCAs can activate neutrophils. However, ANCA pathogenicity is dependent on both affinity and epitope specificity, and there also seem to be ANCAs that are non-pathogenic and even beneficial. NETs are most probably formed at a constant rate in healthy individuals, but NET formation can become highly elevated by infections, certain drugs, and possibly by epigenetic changes as one age. Increased NET formation must be balanced by clearance mechanisms, which seem to include DNaseI and possibly autoantibodies with ANCA specificity. We hypothesize that under unfavorable circumstances some individuals (partly depending on genetics) develop pathogenic autoantibodies that can activate neutrophils thus creating a vicious circle resulting in widespread vessel wall inflammation.

## Author Contributions

DS has written most of the text, but in close collaboration with MS.

## Conflict of Interest Statement

The authors declare that the research was conducted in the absence of any commercial or financial relationships that could be construed as a potential conflict of interest.
